# Humoral immune responses and adverse effects of inactivated West Nile virus and tetanus toxoid vaccines administered via the subcutaneous or intramuscular route in horses

**DOI:** 10.3389/fvets.2026.1931255

**Published:** 2026-10-14

**Authors:** Zuzanna M. Pietras, Macarena G. Sanz, Erin Pinnell, Ana Rangel, Debra C. Sellon

**Affiliations:** 1 Department of Clinical Sciences, College of Veterinary Medicine, Kansas State University, Manhattan, KS, United States; 2 Department of Veterinary Clinical Sciences, College of Veterinary Medicine, Iowa State University, Ames, IA, United States; 3 Department of Veterinary Clinical Sciences, College of Veterinary Medicine, Washington State University, Pullman, WA, United States; 4 Department of Large Animal Clinical Sciences, Virginia Maryland College of Veterinary Medicine, Blacksburg, VA, United States

**Keywords:** horse, immune-mediated myositis, intramuscular, myosin heavy chain myopathy, subcutaneous, tetanus, vaccine, West Nile virus

## Abstract

**Introduction:**

Myosin heavy chain myopathy (MYHM) is a genetic disorder in Quarter Horse-related breeds that can result in non-exertional rhabdomyolysis and immune-mediated myositis. Infectious diseases and muscle damage, particularly from intramuscular (IM) vaccination, have been implicated as potential triggers for this condition. However, the subcutaneous (SC) injection route has not been investigated as an alternative vaccination method for MYHM-affected horses. The hypothesis was that there would be no difference in humoral immune responses or injection site reactions in healthy horses receiving killed West Nile virus (WNV) and tetanus toxoid vaccines via SC and IM routes. The primary objective was to determine serum antibody titers and the adverse effects elicited by both vaccination routes.

**Methods:**

A randomized, single-blind, two-arm, two-period study compared the responses in horses receiving WNV and tetanus vaccines via IM or SC routes. A total of 17 adult mares, all of which were negative for the MYH1 mutation, from a university research and teaching herd were included in the study. Serum antibody levels were assessed on the day of each vaccination. Antibody levels were also assessed on days 28 and 56 after WNV vaccine and on day 28 after tetanus toxoid administration. Vaccine site reactions were assessed daily for 72 h after vaccination. Antibody levels and injection site reactions were analyzed using two-way repeated measures ANOVA, rank sum tests, and Fisher’s exact test, with statistical significance at a *p*-value of <0.05.

**Results:**

Serum neutralization antibody titers increased after WNV vaccination regardless of the route of vaccine administration (*p* = 0.007), with no differences observed between routes of administration. Injection site swelling was greater after SC vaccination than after IM injection route for the WNV vaccine (*p* = 0.003). Baseline tetanus titers were high in almost all horses, and no change in titer was observed following vaccination. Injection site swelling was greater after SC tetanus vaccination than after IM vaccination (*p* = 0.002).

**Discussion:**

Subcutaneous vaccination was associated with significant local reactions. An accurate assessment of humoral responses was challenging as the horses included in the study had high baseline titers from previous vaccinations. As a result, drawing definitive conclusions regarding the equivalence of responses to SC versus IM vaccination is difficult. Therefore, the results regarding this comparison must be considered inconclusive.

## Introduction

The American Quarter Horse is the most common horse breed in the United States (1). A variety of inherited disorders, many of which affect muscle function, have been identified in this breed (2). The American Quarter Horse Association (AQHA) requires horses to undergo genetic testing for hyperkalemic periodic paralysis (HYPP), polysaccharide storage myopathy (PSSM), malignant hyperthermia (MH), glycogen branching enzyme deficiency (GBED), hereditary equine regional dermal asthenia (HERDA), and myosin heavy chain myopathy (MYHM) before registration in an effort to reduce the prevalence of clinically affected animals and limit the number of carriers of these diseases, which are inherited in an autosomal recessive pattern (3, 4).

Myosin heavy chain myopathy (MYHM) is a novel genetic disorder that has recently been described in Quarter Horses and related breeds, including Appaloosas and American Paint Horses. This condition is associated with a single segregating non-synonymous E321G mutation in the MYH1 gene, which encodes the myosin-type 2X heavy chain (4–6). The E321G MYH1 variant allele is present in 6.8% of American Quarter Horses, with the highest prevalence among the reining, working cow, and halter performance subgroups (4, 7). Horses carrying this mutation are susceptible to non-exertional rhabdomyolysis (non-ER), immune-mediated myositis (IMM), and systemic calcinosis (calciphylaxis) (4, 6, 8). Clinical signs vary for each syndrome but may include muscle stiffness, weakness, myoglobinuria, and acute and severe muscle wasting. The most significant clinicopathological finding is an increase in serum creatine kinase (CK) activity (6, 9).

Because MYHM is inherited in a codominant manner with variable penetrance, both heterozygous (My/N) and homozygous (My/My) animals can be affected. Homozygous horses tend to have more severe clinical signs, a higher recurrence rate, and an overall poorer prognosis (4, 6, 9). The exact pathogenesis of IMM and non-ER is unclear, but in horses with IMM, the affected muscle is characterized histologically by lymphocytic infiltrates in type 2X myofibers (5, 6).

Initial research suggested that clinical signs of IMM may be triggered by exposure to an infectious agent or by vaccination (4, 6). Some experts recommend avoiding vaccination of horses with this genetic mutation or using only the most essential monovalent vaccines (10). It is unknown whether muscle trauma from an intramuscular (IM) injection or the immune response triggered by vaccination may be the inciting cause of IMM. Therefore, using subcutaneous (SC) vaccination rather than the approved IM route might decrease the likelihood of triggering IMM in susceptible horses.

Subcutaneous administration of vaccines is common in veterinary species other than horses. There are vaccines available for horses that are approved for SC administration. One example is Tetguard® tetanus toxoid (Boehringer Ingelheim, Inc.).

The hypothesis for this study was that there is no difference in humoral immune responses or injection site reactions in healthy adult mares receiving inactivated West Nile virus vaccine and tetanus toxoid vaccine via the SC route compared to the IM route. The study objectives were (1): to determine whether humoral immune responses elicited by SC administration of killed West Nile virus and tetanus toxoid vaccines are equivalent to responses elicited by IM administration of the same vaccines in mares, and (2) to determine whether SC administration of these vaccines is associated with an increased incidence of adverse effects compared to IM administration.

## Materials and methods

### Study design

This study used a randomized, single-blind, two-arm, two-period design to compare humoral immune responses and adverse effects in horses receiving WNV and tetanus vaccines via SC or IM routes. An overview of the experimental protocol is shown in Figure 1. The sample size was calculated based on the anticipated adverse effect of visible swelling. Based on clinical experience, IM injection was expected to result in minimal detectable swelling compared to SC injection. Sample size calculations used a mean measurable swelling of 0 cm ± 1 cm for IM injections and 2.5 cm (1 inch) ± 5 cm for SC injections. To detect a difference with an alpha of 0.05 and a statistical power of 80%, a sample size of at least 6 horses per group was required.^1^

**Figure 1:**
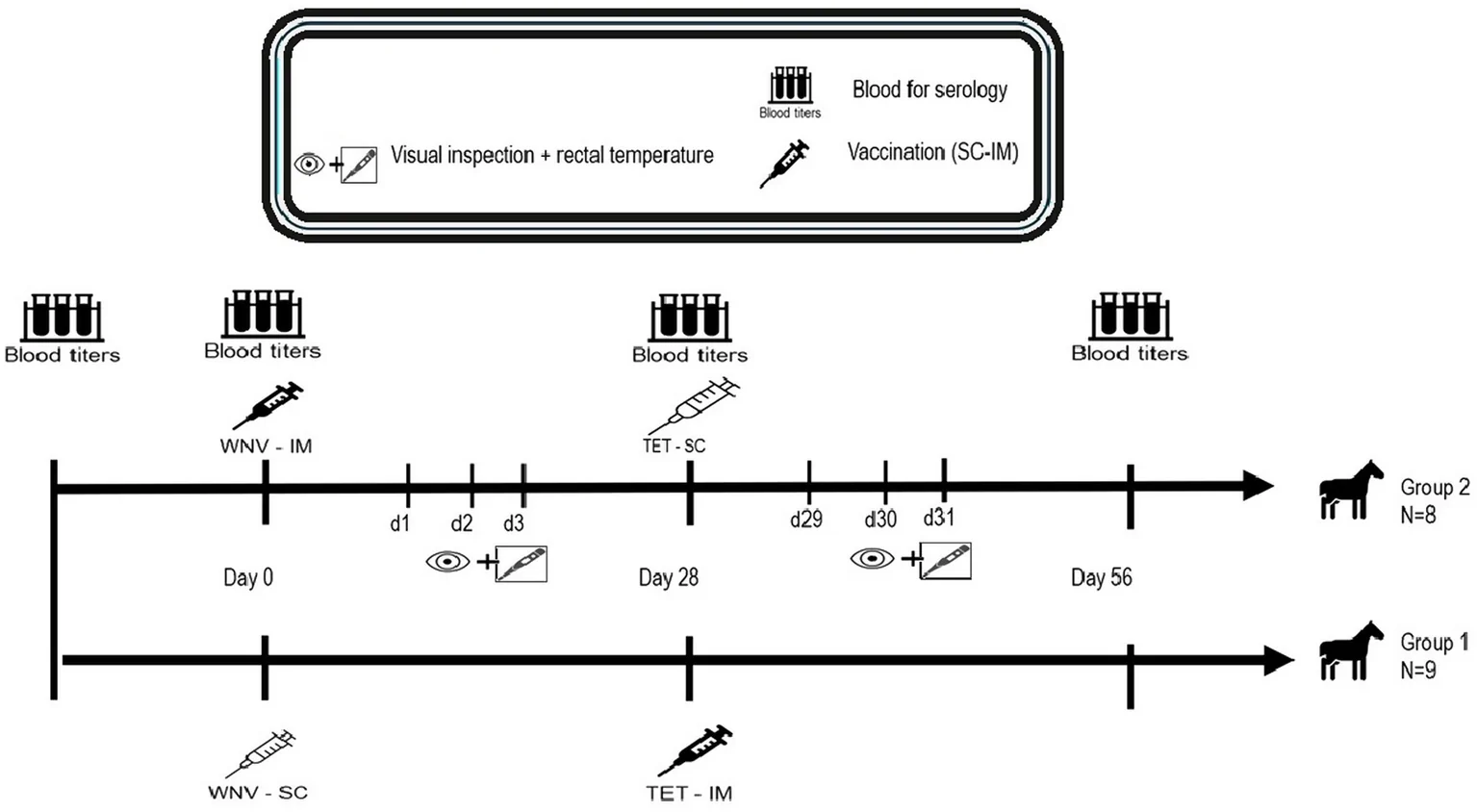
Diagram illustrating the experimental protocol. WNV, West Nile virus; IM, intramuscular; TET, tetanus; SC, subcutaneous.

### Animals

A total of 17 systemically healthy adult mares (16 American Quarter horses and 1 Thoroughbred) from a university research and teaching herd were included in the study. The mean age of the horses was 14.1 ± 2.0 years (range = 9–17 years). The mean body weight was 542 ± 49 kg (range = 430–620 kg). All horses tested negative for the presence of the MYH1 mutation (Veterinary Genetics Laboratory, UC Davis, CA, USA). All mares were previously vaccinated with the Prestige 5 Way + WNV® vaccine (Eastern encephalomyelitis, Western encephalomyelitis, rhinopneumonitis [EHV-1 and EHV-4], influenza [including H3N8 strains EIV A/eq/Florida/RW/13, EIV A/equine 2/Kentucky/02 American, EIV A/eq/Richmond/1/07], West Nile virus, and tetanus toxoid; Zoetis, Inc.) with an annual booster each spring for several years prior to this study.

### Antibody measurement

Day 0 was defined as the day of initial WNV vaccine administration. The timing of peak antibody responses after vaccination varies greatly between individuals, but a significant increase in antibody concentrations is expected at approximately 4 weeks (11). Blood was collected through jugular venipuncture for WNV antibody assays on days 0, 28, and 56 and for tetanus titers on days 28 and 56. At each time point, 10 mL of blood was collected into a plain red-top Vacutainer® tube. Blood was centrifuged, and the serum was apportioned into 1 mL aliquots and frozen at −20 °C until the antibody titers were analyzed.

West Nile virus IgM and IgG antibody levels were assessed using capture enzyme-linked immunosorbent assays (ELISAs), which are specific to immunoglobulin isotypes. The results were reported as a positive-to-negative ratio (PNR), calculated as the mean optical density with WNV antigen divided by the mean optical density with control antibody, and expressed as a dimensionless index rather than an endpoint antibody titer. The West Nile virus IgG titer was also assessed using a serum neutralization (SN) assay. All WNV assays were performed at the Animal Health Diagnostic Center of Cornell University (Cornell, NY, USA).

Baseline tetanus immunoglobulin titers were assessed in the Cornell University research laboratory of Dr. Bettina Wagner. After randomization, baseline titers for each group were assessed and confirmed to have comparable baseline geometric mean titers.

### Vaccine administration

Mares were vaccinated with Vetera West Nile Virus® vaccine (killed virus, Boehringer Ingelheim, Inc., Duluth, GA, USA) on day 0 and with Tetguard Tetanus Toxoid® vaccine (Boehringer Ingelheim, Inc., Duluth, GA, USA) on day 28. Group 1 mares (*n* = 9) were vaccinated subcutaneously in the neck region with the WNV vaccine and intramuscularly with tetanus toxoid. Group 2 mares (*n* = 8) were vaccinated intramuscularly with WNV vaccine and subcutaneously with tetanus toxoid. Both IM and SC injections were performed in the neck region, avoiding the cervical vertebrae, nuchal ligament, and scapula. Intramuscular injections were performed in the cervical serratus ventralis muscle. Subcutaneous injections were performed in the SC tissue overlying that same muscle. Injection sites were clipped, cleaned with isopropyl alcohol, and marked with a permanent marker (Sharpie®, Newell Brands, Atlanta, GA, USA). One mL of each vaccine was injected using a 20-gauge, 1-inch hypodermic needle.

### Monitoring

Mares were monitored daily for 72 h after each injection by a blinded observer (ZMP). Monitoring included assessing attitude and appetite, rectal temperature, total area of injection-site swelling measured using calipers, and pain responses to manual palpation of the injection site. Pain responses to manual palpation were scored as 0 (no pain), 1 (very mild pain), 2 (mild pain), or 3 (moderate to severe pain). All pain assessments for all horses were performed by the same blinded observer. Serum amyloid A (SAA) concentrations were measured before each vaccination and 24 h after each vaccination using a commercially available stall-side assay (VMRD, Pullman, WA, USA). The size of the visible swelling at the injection site on each horse was calculated as the product of the length and width of the swollen area.

### Statistical analysis

Data were analyzed using SigmaPlot software (Grafiti, Inc., Palo Alto, CA). Data were assessed for normality and equal variance using the Shapiro–Wilk test. Pre- and post-vaccination antibody levels were compared using a two-way repeated-measures analysis of variance (ANOVA) followed by a Bonferroni *t*-test for *post hoc* evaluation. Serum neutralization titers for WNV were analyzed as raw data and after log base 2 transformation. Sizes of post-vaccination swelling at 48 h after SC administration of each vaccine type were compared using a rank sum test. The difference between baseline and 48 h was chosen for comparison because 48 h was the time when reactions were subjectively assessed to be most relevant. The presence or absence of pain and swelling after vaccination was compared between IM and SC administration for each vaccine type (WNV and tetanus) using a Fisher’s exact test. For all analyses, statistical significance was defined as a *p*-value of <0.05.

## Results

### Humoral immune responses

Capture ELISA assays revealed that IgM antibody levels to WNV were low for all horses at time 0, and no significant changes occurred over time after either IM or SC vaccination (Figure 2), indicating that no natural exposure occurred immediately before or during the study period. Capture ELISA IgG values for WNV did not increase over time after either IM or SC vaccination, but there was considerable individual variation (Figure 3). Values did increase substantially over time in two horses [horse 1–4 (SC vaccination) and horse 2–6 (IM vaccination)]. In a third horse (horse 2–8, IM vaccination), values increased from a negligible baseline. Another horse (horse 2–5, IM vaccination) had a very low baseline value with no demonstrable increase in value over time. There were no differences in SN titers between IM and SC vaccination. Regardless of the administration route, SN titers for WNV were significantly increased at 28 days after WNV vaccination (*p* = 0.007) as compared to time 0. The results were similar when analysis was repeated after log base 2 transformation of the titers (*p* = 0.011) (Figure 4).

**Figure 2:**
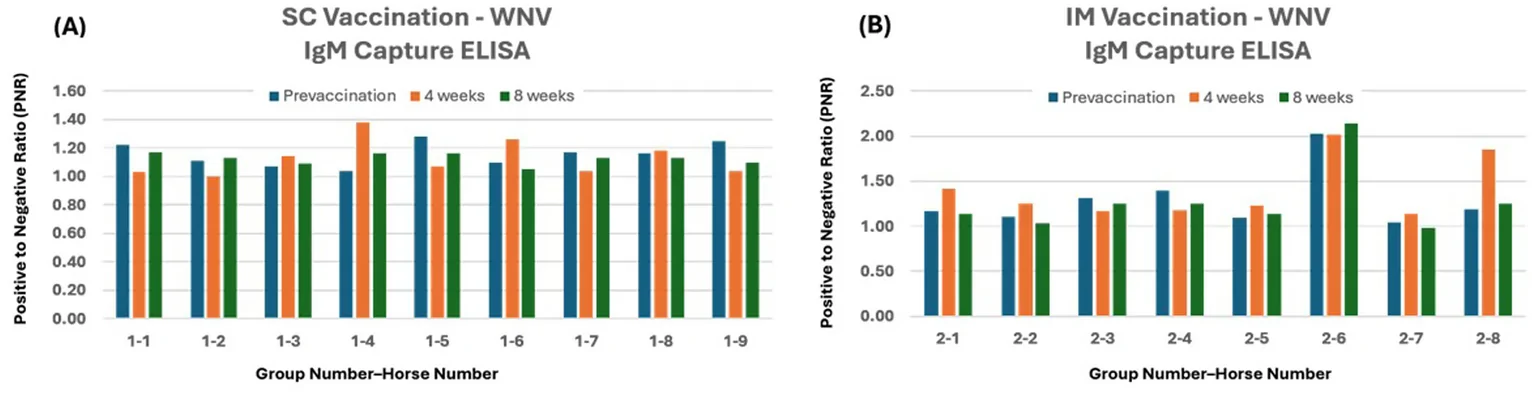
West Nile virus (WNV) IgM capture enzyme-linked immunosorbent assay (ELISA) titers on days 0, 28, and 56 after subcutaneous (SC, panel **A**) and intramuscular (IM, panel **B**) administration of WNV vaccine. The y-axis ranges differ between panels because of the very high baseline value for horse 2–6. Values represent the calculated PNR for each sample (see text for details).

**Figure 3:**
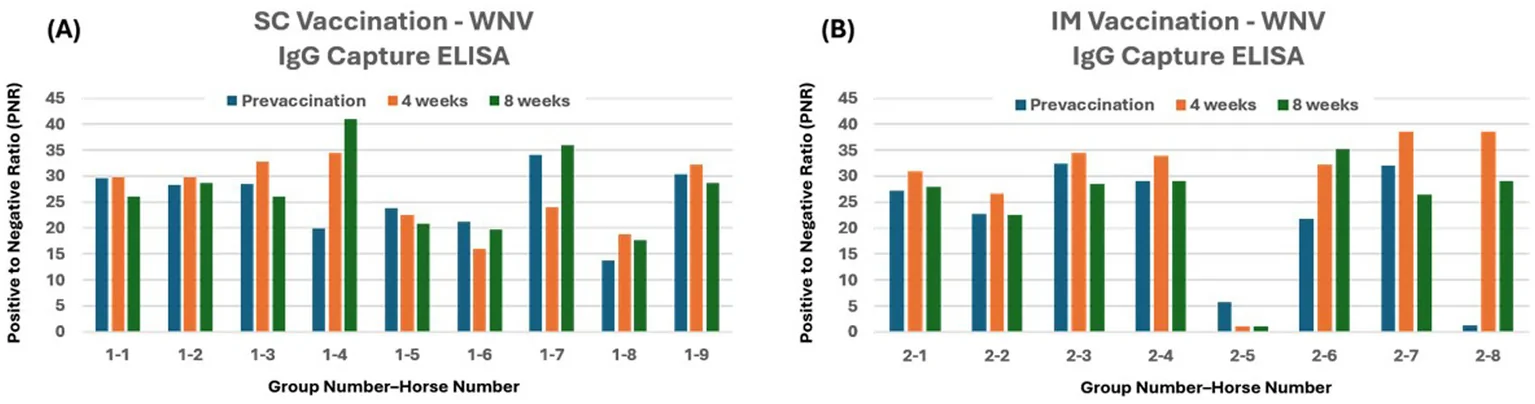
West Nile virus (WNV) IgG capture enzyme-linked immunosorbent assay (ELISA) titers on days 0, 28, and 56 after subcutaneous (SC, panel **A**) and intramuscular (IM, panel **B**) administration of WNV vaccine. Values represent the calculated PNR for each sample (see text for details).

**Figure 4:**
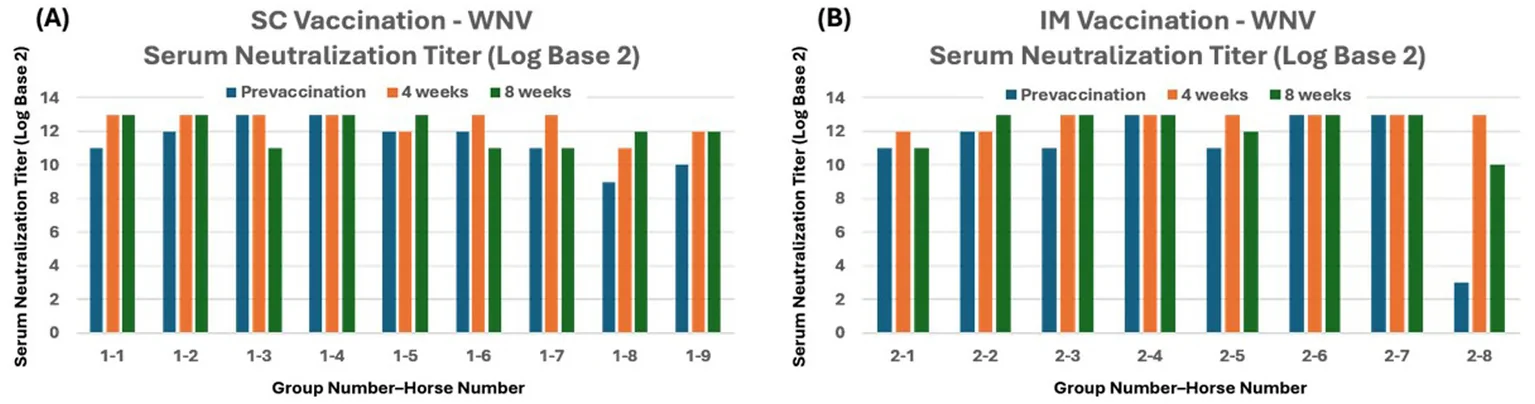
West Nile virus (WNV) serum neutralization (SN) titers on days 0, 28, and 56 after subcutaneous (SC, panel **A**) and intramuscular (IM, panel **B**) administration of WNV vaccine.

All horses had high total anti-tetanus immunoglobulin titers on day 0. There was no significant difference in titers between horses vaccinated by IM or SC routes, and total immunoglobulin titers did not increase in either group after vaccination (*p* = 0.09; Figure 5).

**Figure 5:**
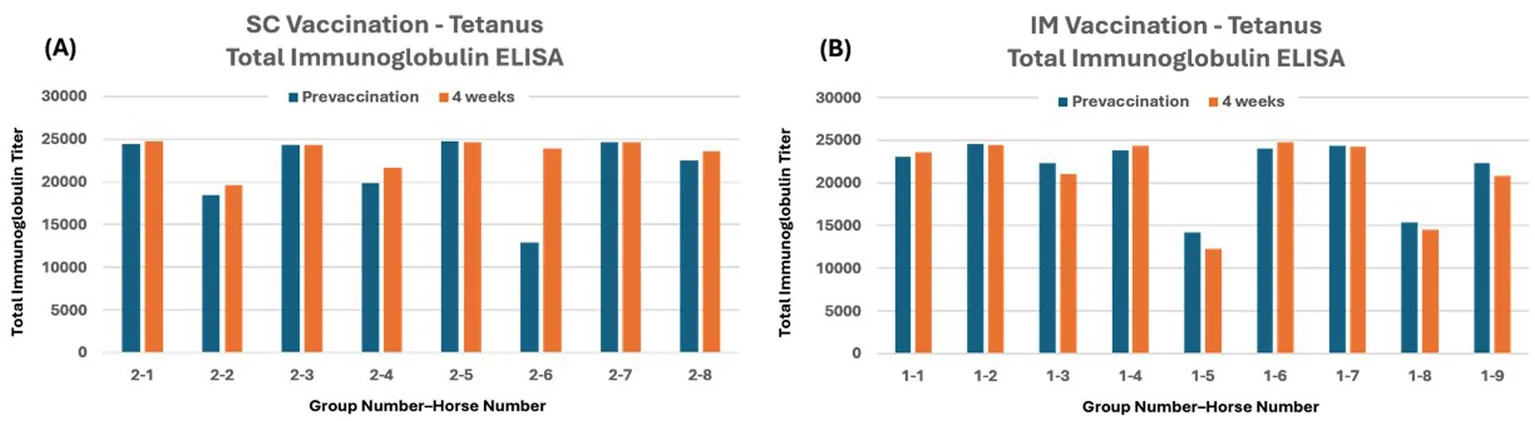
Total anti-tetanus immunoglobulin enzyme-linked immunosorbent assay (ELISA) titers determined from serum samples obtained on days 28 and 56 of the protocol. Day 28 of the protocol was the date of tetanus toxoid administration, and those values represent baseline pre-vaccination tetanus titers. Tetanus toxoid was administered either subcutaneously (SC, panel **A**) or intramuscularly (IM, panel **B**). Post-vaccination titers were determined using serum samples obtained at 28 days after tetanus toxoid administration (day 56 of the protocol).

### Adverse effects

None of the horses developed lethargy or anorexia during the monitoring period. There were no significant changes in heart and respiratory rates. Two horses in group 1 and two horses in group 2 developed a mild fever (rectal temperatures between 38.7 °C and 38.8 °C) on day 1 after WNV vaccination, which was self-limiting and resolved by day 2. No horses developed a fever after tetanus toxoid administration.

Pain scores were assessed daily before WNV vaccine administration and at 24, 48, and 72 h after vaccination. Prior to vaccination, all horses exhibited no pain upon manual palpation of the neck. After vaccination, very mild to moderate pain was detected with manual palpation of the injection site in six of nine horses (67%) that received SC vaccinations. In the first 72 h after vaccination, no pain was detected on manual palpation of the injection site of any horses that received IM WNV vaccination (*p* = 0.009). Similarly, no pain was detected on manual palpation of the injection site in any horses prior to tetanus toxoid administration. In the first 72 h after vaccination, very mild to moderate pain was detected at least once in five of eight horses (62.5%) that received SC vaccination, compared to just one of nine horses (11.1%) that received IM tetanus toxoid (*p* = 0.05).

Injection site swelling was more frequent (*p* = 0.05) and larger (*p* = 0.003) after SC WNV vaccination, compared to IM vaccination sites (Table 1). The frequency and size of injection site swelling were also greater (*p* = 0.03 and *p* = 0.002, respectively) after SC administration of tetanus toxoid, compared to IM administration of the same vaccine. There was no significant difference in swelling size between the SC WNV and the SC tetanus toxoid injection on day 2 (*p* = 0.4). Despite significant pain and swelling after SC administration of both vaccines, physical examinations revealed no signs of abscess formation at the injection site in any of the horses during the monitoring period.

**Table 1:** Median area measurements with interquartile range (IQR, mm^2^) of injection site swelling on day 2 after specified vaccine administration.

Vaccine	Injection route	Number (%) of horses with visible swelling	Median (IQR) swelling size (mm^2^)	*p*-value
Inactivated WNV	SC	8/9 (88.9%)	8,467 (2738–10,609)	0.05*
	IM	3/8 (37.5%)	0 (0–261)	0.003^+^
Tetanus toxoid	SC	8/8 (100.0%)	3,367 (1951–9,579)	0.03*
	IM	4/9 (44.4%)	0 (0–1,303)	0.002^+^

WNV, West Nile virus; SC, subcutaneous; IM, intramuscular. ^*^Indicates *p*-value for proportions of horses with visible swelling after the designated type and route of vaccination. ^+^Indicates *p*-value for size of swelling observed after the designated type and route of vaccination.

Serum amyloid A concentrations were below the detection threshold (<20 μg/mL) in all horses on the day of WNV vaccination. Serum amyloid A concentrations increased after WNV vaccine administration, regardless of whether it was given subcutaneously or intramuscularly, with no detectable difference in the magnitude of increase between the two routes (Table 2). All horses had SAA concentrations of <20 μg/mL on the day of tetanus toxoid administration. At 24 h after tetanus vaccination, SAA concentrations remained below the detection threshold for all horses, regardless of the route of vaccine administration.

**Table 2:** Median and interquartile range (IQR) of serum amyloid A concentration (SAA, μg/mL) at 24 h after inactivated WNV vaccine administration by the specified route of injection.

Vaccine	Injection route	Number (%) of horses with SAA >20 μg/mL	Median (IQR) SAA (μg/mL)	*p*-value
Inactivated WNV	SC	6/9 (66.7%)	107 (0–254)	0.6
	IM	7/8 (87.5%)	93 (52–279)	

All horses had SAA concentrations <20 μg/mL on the day of vaccination. WNV, West Nile virus; SC, subcutaneous; IM, intramuscular.

Seven of nine horses that received the SC WNV vaccine (77.8%) were treated with non-steroidal anti-inflammatory drugs (NSAIDs) administered systemically [phenylbutazone 2.2–4.4 mg/kg by mouth, once to twice daily (VetriBute, VetOne®, Austin, TX, USA)] and topically [1% diclofenac sodium cream (Surpass®, Boehringer Ingelheim, Inc., Duluth, GA, USA)] due to significant injection site swelling and pain. No horses required analgesic or anti-inflammatory treatment after IM administration of this vaccine. None of the horses required treatment following vaccination with tetanus toxoid, regardless of the route of administration.

## Discussion

The results of this study did not demonstrate significant differences in humoral immune responses related to the route of WNV or tetanus vaccine administration, but significant differences in adverse effects were observed. Systemic adverse effects were minor and limited primarily to mild fever after WNV vaccine administration, which was observed regardless of the route of injection. In contrast, injection site swelling was more frequent, larger in size, and lasted longer after SC administration, compared to IM administration.

West Nile virus and tetanus toxoid vaccines were used in this study as these vaccines are commonly recommended as minimum immunizations for horses that are prone to vaccine reactions. IMM has been reported to be more commonly triggered by influenza, equine herpes virus 4, and/or strangles vaccines; therefore, vaccinations for these diseases should be considered in future studies (6).

The results of IgM capture ELISA suggest the absence of natural exposure to WNV during the study period. While IgM antibodies have been reported to be increased for up to 42 days after the administration of the inactivated WNV vaccine (12), no detectable IgM response was observed in our study.

The majority of the horses in this study had very high baseline anti-tetanus Ig and WNV SN titers, with many values at or very close to maximum values reportable by the diagnostic laboratory. These high baseline titers were most likely attributable to previous annual vaccinations. Even though there was a statistically significant increase in WNV SN titers after both SC and IM vaccination, this difference may not be biologically significant given the relatively high baseline titers in the majority of horses. Future studies might use naïve, unvaccinated horses to accurately compare humoral immune responses after initial vaccination.

Because protection against both WNV and tetanus has been linked to humoral immune responses, high antibody levels are likely to indicate the presence of a protective immune response. Although the antibody levels required for protective immunity against WNV or tetanus in horses have not been definitively determined, the American Association of Equine Practitioners has made suggestions to assist with this type of assessment. It is possible that antibody measurements might be considered as an alternative to routine booster vaccinations in horses prone to IMM or other severe vaccine reactions (13). This study also identified one horse that did not respond adequately to WNV vaccination (horse 2–5, IM vaccination). Serology can be a useful tool in identifying those non-responders.

Injection site reactions were very common in the horses in this study after SC administration of vaccines. The total area of the local swelling after SC administration of either WNV or tetanus vaccine was significantly larger than that observed after IM administration of the same vaccines. This degree of swelling and the frequency of site pain observed after SC vaccine administration will likely limit the acceptability of this route of vaccine administration for many horse owners. The visible inflammation was primarily observed in subcutaneous tissues after SC vaccination; however, potential inflammatory changes in the underlying muscles were not critically evaluated. It is possible that muscles experienced increased inflammation after SC injection, raising the possibility that the markedly greater local inflammatory response might actually be detrimental for horses with the MYHM mutation. Serum creatine kinase activities were not measured before and after vaccination in this study. It is possible that such measurements might have provided additional information regarding the extent of local muscle damage after vaccination.

Serum amyloid A concentrations were measured before vaccine administration and 24 h after vaccination to assess the magnitude of systemic inflammation. Serum amyloid A is an acute-phase protein that increases rapidly during inflammation and decreases rapidly as inflammation resolves, making it a sensitive but non-specific marker of acute-phase responses (14, 15). It has been reported to increase within 12–24 h following routine vaccination in horses, and to return to normal values by day 9 post-vaccination (16, 17). In this study, SAA levels increased slightly after WNV vaccination, regardless of the route of vaccine administration. Tetanus toxoid administration was not associated with increased SAA concentrations after either SC or IM administration, suggesting that this vaccine may not induce a significant systemic inflammatory response.

The use of non-steroidal anti-inflammatory drugs (NSAIDs) to decrease vaccine-associated pain and inflammation may have decreased immune responses to the vaccine, as previously suggested for influenza vaccination in horses (18). However, the supporting study included only a small number of horses. Studies in human medicine have been contradictory but suggest that NSAIDs have only minor effects on most vaccine responses (19, 20). It is possible that the use of NSAIDs in the horses with severe local inflammation in this study may have impaired immune response to vaccination, but this could not be determined with the available data.

The limitations of this study include a relatively small sample size, limited gender and breed of horses, use of previously vaccinated horses limiting the ability to assess humoral immune responses, inability to assess cellular immune responses, lack of measurements of serum creatine kinase activities, and only using horses negative for the MYH1 mutation. Increases in SN titers to WNV were demonstrable after either SC or IM administration of WNV vaccine, but that route of administration was associated with significant local adverse effects, which limit its suitability for use in many horses. Determining antibody titers expected to protect horses against WNV or tetanus would likely be of great benefit in decision-making related to vaccination of horses at high risk of severe vaccine reactions.

## Data Availability

The raw data supporting the conclusions of this article will be made available by the authors, without undue reservation.

## Footnotes

1
https://epitools.ausvet.com.au
